# Neuroanatomical stratification of 12-months suicidal risk in bipolar disorder with XBoost

**DOI:** 10.1192/j.eurpsy.2026.10214

**Published:** 2026-07-31

**Authors:** A. Pigoni, I. Tesic, S. Peri, G. Delvecchio, C. Pini, C. Moltrasio, N. Citerà, G. Torino, L. Squarcina, P. Brambilla

**Affiliations:** 1 IRCCS Fondazione Ca Granda; 2 University of Milan, Milan, Italy

## Abstract

**Introduction:**

**Predicting suicidal behaviors in patients with bipolar disorder (BD)** is an unmet challenge due to the **absence of biomarkers** [1]. Creating meaningful risk subgroups, by stratifying patients based on their individual risk, could greatly assist clinicians in decision-making processes.

**Unsupervised and semi-supervised machine learning (ML) techniques are valuable tools for dissecting the heterogeneity of psychiatric diagnosis** [2].

**Objectives:**

The aim of the study is the definition of **risk subgroups among individuals with BD, to uncover clusters that correlate with 12-months suicide risk**, using neuroanatomical features (grey matter (GM) volumes) from 136 brain areas (Neuromorphometrics atlas) [3]

**Methods:**

We enrolled 163 individuals with BD (53% females, mean age 44.8, SD 15.3) during an acute admission.

At baseline, clinical and demographic data were obtained; moreover, patients underwent MRI scan and were **then followed-up for 12 months.**

A semi-supervised machine learning model was trained using the XGBoost algorithm on the GM volume features, employing a repeated 5-fold cross-validation. To address class imbalance between suicide attempters and non-attempters, the ROSE (Random Over-Sampling Examples) technique was applied to create a synthetic balanced dataset integrating over-sampling of the minority class and under-sampling of the majority class.

Model-derived probabilities of suicide attempts in the follow-up were used for soft clustering, stratifying patients into three risk categories: Low (P ≤ 0.1), Medium (0.1 < P ≤ 0.93), and High-Risk (P > 0.93).

Post-hoc comparisons across clusters were conducted to explore intergroup clinical differences.

**Results:**

During the 12-months observation, **10 patients (6.13%) attempted suicide**.

**The model stratified our sample in a 3-clusters of low, medium and high risk** for attempting suicide in the 12-months follow-up. The final model achieved an AUC of 0.90, with 90% sensitivity and 71% specificity, and an overall accuracy of 72.2%. Low-risk cluster includes 66 subjects and no suicide attempters; the Medium-risk cluster consists of 76 subjects, with one suicide attempt; finally, **the High-risk cluster is composed of 20 subjects, 9 of which attempted suicide**.

High-Risk patients were significantly younger than those in other groups and showed a higher number of psychiatric contacts. They reported the highest number of previous suicide attempts, especially within the last 12 months. They had significantly lower chlorpromazine equivalents, lower lithium use, and higher use of antiepileptics.

**Conclusions:**

We successfully identified a **three-cluster model based on neuroanatomical features.** The clusters showed different levels of suicide risk and presented significant differences in clinical characteristics. If confirmed in independent samples, these findings hold potential to inform clinicians’ decision-making, facilitating personalized risk stratification.

**Disclosure of Interest:**

None Declared

